# Ein typisches myofasziales Schmerzsyndrom, oder doch nicht? Seltene Differenzialdiagnose

**DOI:** 10.1007/s00108-025-02029-6

**Published:** 2025-12-17

**Authors:** Ference van den Bos, Juliane Franz

**Affiliations:** 1 Klinik für Rheumatologie, Universitätsspital Zürich, Zürich, Schweiz; 2 Rheumatologie und Rehabilitation, Kantonsspital Baden, Baden, Schweiz

## Abstract

Ein 55-jähriger Patient stellte sich vor mit generalisierten myofaszialen Beschwerden. Die klinischen und Laboruntersuchungen waren unauffällig bis auf eine gespannte Haut an den unteren Extremitäten und eine Bluteosinophilie. Bei Anreicherung der Faszien in der Magnetresonanztomographie (MRT) und Nachweis entzündlicher epifaszialer Infiltrate in der Muskelbiopsie wurde die Diagnose einer eosinophilen Fasziitis gestellt. Unter Glukokortikoiden, Methotrexat und Tocilizumab kam es zur vollständigen klinischen und bildmorphologischen Regredienz der Fasziitis.

## Abstract (en)

A 55-year-old male patient presented with generalized myofascial complaints. The clinical and laboratory investigations were unremarkable apart from taut skin of the lower extremities and eosinophilia of the blood. Based on the enrichment of the fascias seen in magnetic resonance imaging (MRI) and the detection of inflammatory epifascial infiltrates in the muscle biopsy, the diagnosis of eosinophilic fasciitis was made. Treatment with glucocorticoids, methotrexate and tocilizumab led to a complete clinical and morphological regression of the fasciitis.

## Hintergrund

Die eosinophile Fasziitis (EF), auch Shulman-Syndrom genannt, ist eine sehr seltene, fibrosierende Erkrankung der Faszien [1]. Typischerweise treten Erytheme, Ödeme und Indurationen an den Extremitäten auf. Charakteristische Befunde wie das „groove sign“ und die Peau-d’orange*-*Hautveränderung können fehlen, weshalb die Erkrankung oft nicht erkannt wird. Glukokortikoide werden als First-line-Therapie eingesetzt, für die längerfristige Therapie gibt es bisher keine evidenzbasierten Empfehlungen.

## Anamnese

Ein 55-jähriger Patient wurde uns zugewiesen aufgrund von generalisierten Myalgien, die vier Monate zuvor nach einer Hüft-Totalendoprothese-Implantation links begonnen hatten. Er gab vor allem „brennende“ Schmerzen in den Beinen und den Oberarmen an, mit einem Schmerzmaximum am Morgen, begleitet von einer Schwellung und einem Spannungsgefühl. Eine neurologische Abklärung inklusive klinischer Untersuchung und Elektroneurographie und Elektromyographie (ENMG) war unauffällig.

Anamnestisch sind degenerative Erkrankungen des Bewegungsapparats, eine arterielle Hypertonie, eine Hypercholesterinämie, eine bipolare Störung sowie eine aktuelle psychosoziale Belastungssituation zu erwähnen. Die Medikation bestand aus einem Antihypertensivum, Lithium seit vielen Jahren und neu aus Atorvastatin.

## Befunde

Die klinische Untersuchung war unauffällig bis auf ein diffus druckempfindliches Weichteilgewebe der Extremitäten sowie eine auffällig straffe, nicht abhebbare Haut mit fehlender Hautfaltenbildung ohne Rötung und ohne Eindellungen oder Furchenbildungen an den Unterschenkeln. Die ersten Laboruntersuchungen einschließlich C-reaktiven Proteins (CRP) und Autoantikörperdiagnostik (inklusive antinukleären Antikörper [ANA]) waren unauffällig bis auf eine leicht erhöhte Kreatinkinase (287 U/l, Referenzbereich < 190 U/l), die bei Kontrolle wieder normwertig war.

Bei Fehlen richtungsweisender Befunde und unter Berücksichtigung der damaligen psychosozialen Belastungssituation des Patienten erwogen wir zunächst ein myofasziales Schmerzsyndrom – eine chronische Schmerzerkrankung mit lokalen druckschmerzhaften muskulären Verhärtungen (Triggerpunkten) und teils ausstrahlenden Schmerzen, häufig ausgelöst durch Fehlhaltung, Überbelastung und Stress. Eine medikamentös bedingte Ursache der Myalgien durch Atorvastatin oder Lithium erschien uns weniger wahrscheinlich, da die Beschwerden bereits vor Beginn der Therapie mit Atorvastatin aufgetreten waren und der Patient Lithium schon seit vielen Jahren unverändert einnahm.

Auffällig im Verlauf war jedoch eine neu aufgetretene periphere Eosinophilie (3,35 Tsd./µl bzw. 25 %, Referenzbereich 0–0,6 Tsd./µl bzw. < 5 %) bei normalem IgE-Spiegel, fehlendem Asthma bronchiale oder einer allergischen Diathese. Auch lag eine polyklonale Hypergammaglobulinämie vor.

Da weder klinische Hinweise auf eine infektiöse Erkrankung noch eine auffällige Reiseanamnese oder ein intensiver Tierkontakt vorlagen und der IgE-Spiegel normal war, wurde von einer weiterführenden infektiologischen Abklärung der Eosinophilie abgesehen.

In Zusammenschau mit den Myalgien ergab sich die Verdachtsdiagnose einer eosinophilen Fasziitis.

**Abb. 1:**
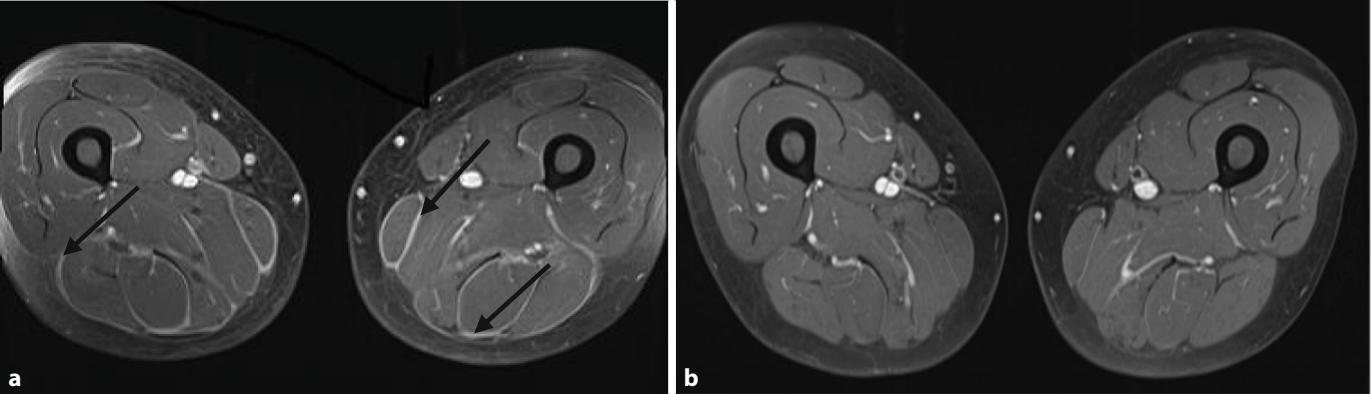
MRT-Untersuchung der unteren Extremitäten (T1-Sequenz mit Kontrastmittel). **a** Der wichtigste MRT-Befund bei EF-Patienten ist die Verdickung der Muskelfaszien mit hoher Signalintensität oder Kontrastverstärkung der Faszien, wobei die angrenzende Muskulatur und das subkutane Fettgewebe meist verschont bleiben. Schwarzer Pfeil: Kontrastmittelanreicherung der Faszien. **b** Komplette Regression der Signalintensität der Faszien nach Beginn der Therapie

Eine MRT-Untersuchung aller Extremitäten zeigte eine symmetrische Verdickung und Kontrastmittelanreicherung in den Faszien (Abb. 1a), vereinbar mit einer Fasziitis.

**Abb. 2:**
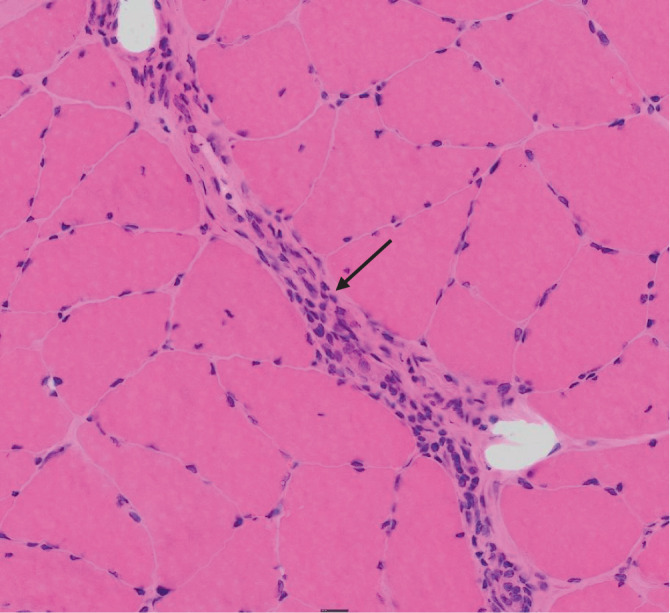
Tiefe Muskelbiopsie mit Hämatoxylin-Eosin-Färbung des M. semitendinosus mit perimysialen Infiltrationen von Lymphozyten. Keine Myositis, keine Vaskulitis

Ergänzend wurde eine tiefe Muskelbiopsie veranlasst. Es zeigten sich fasziendurchsetzende, T‑Zell-dominierte lymphozytäre Infiltrate, vorzugsweise entlang perimysialer Strukturen. Eosinophile Granulozyten waren nicht nachweisbar (Abb. 2).

## Diagnose

Eosinophile Fasziitis (früher Shulman-Syndrom)

## Therapie und Verlauf

Nach Einleitung einer Therapie mit Prednisolon 60 mg/Tag kam es initial zu einer raschen Besserung der Symptome. Diese kehrten jedoch bei einer Dosisreduktion auf 20 mg Prednisolon/Tag zurück. Zudem traten Nebenwirkungen wie psychische Beschwerden, Anzeichen eines Cushing-Syndroms und *Candida*-Infektionen auf. Unter einer steroidsparenden Therapie mit Methotrexat 20 mg s.c. einmal pro Woche zeigte sich zunächst ein positiver Verlauf, die Prednisolondosis konnte auf 5 mg/Tag reduziert werden. Auch waren die Befunde in der MRT der Extremitäten fast vollständig regredient (Abb. 1b). Unter der Behandlung mit Methotrexat traten vermehrt Nebenwirkungen, einschließlich Übelkeit, oraler Aphthen und Kopfschmerzen, auf, die trotz Dosisreduktion persistierten, sodass die Therapie nach vier Monaten beendet wurde. Eine Therapie mit Azathioprin kam wegen der verringerten Thiopurinmethyltransferase(TPMT)-Aktivität bei dem Patienten nicht infrage. Auf Basis einzelner Kasuistiken entschieden wir uns für eine Off-label-Therapie mit dem Interleukin-6-Rezeptor-Antagonisten Tocilizumab. Bei guter Wirksamkeit und Verträglichkeit dieser Therapie konnte die Steroidtherapie zügig beendet werden. Im Rahmen einer Therapiepause wegen einer Schulteroperation traten erneut Myalgien und eine leichte periphere Eosinophilie auf, sodass der Patient die Tocilizumabtherapie vorerst weiterführt.

## Diskussion

Wir stellen einen Fall einer eosinophilen Fasziitis vor, die beinahe als myofasziales Schmerzsyndrom fehlgedeutet worden wäre, da augenscheinliche charakteristische Befunde fehlten.

Es handelt sich um eine seltene fibrosierende Erkrankung der Faszien mit Erythem, Ödem und Induration der Extremitäten. Meist sind dabei alle 4 Extremitäten betroffen, seltener sind nur die unteren oder oberen Extremitäten involviert. Die Fibrose kann zu Gelenkkontrakturen führen. Charakteristische Befunde wie das „groove sign“ (eine lineare Vertiefung der Haut entlang von Venen durch den Zug der fibrosierten Faszien) und die lokalisierte Peau-d’orange-Hautveränderung können, müssen aber nicht vorhanden sein, wodurch die Diagnosestellung wie in unserem Fall erschwert ist [1].

Differenzialdiagnostisch kommt in erster Linie eine Systemsklerose infrage. Eine Abgrenzung zur Systemsklerose war durch die Aussparung der Hände und Füße, das Fehlen eines Raynaud-Syndroms und weiterer für eine Systemsklerose typischer Befunde sowie durch negative ANA möglich [1, 2]. In Tab. 1 sind die wichtigsten Differenzialdiagnosen in unserem Fall aufgeführt.

**Tab. 1:** Differenzialdiagnosen der eosinophilen Fasziitis

Krankheit	Typische Merkmale	Begründung für Ausschluss in unserem Fall
Systemische Sklerose	Raynaud-Phänomen Sklerodermie mit Sklerodaktylie Organbeteiligung Positive ANA	Kein Raynaud-Phänomen Keine Fingerbeteiligung Keine Organmanifestation ANA negativ
Morphea	Umschrieben sklerotische Hautplaques	Abwesenheit typischer Hautläsionen MRT und Biopsie mit Faszienbeteiligung
Nephrogene systemische Fibrose	Auftreten nach Gadoliniumexposition bei eingeschränkter Nierenfunktion	Normale Nierenfunktion
Medikamentös/toxisch induzierte Fasziitis	Z. B. bei Exposition gegenüber auslösenden Substanzen wie L‑Tryptophan, Carbidopa, Silica, Radiatio	Keine entsprechende Exposition bekannt

Ätiologie und Pathogenese sind weiterhin unklar. Einige auslösende Faktoren sind beschrieben, wobei eine Korrelation mit intensiver körperlicher Anstrengung am häufigsten berichtet wird [1]. Im vorliegenden Fall traf dies jedoch nicht zu.

Eine periphere Eosinophilie kann, muss jedoch nicht vorhanden sein. Diese ist oft vorübergehend und korreliert nicht mit der Krankheitsaktivität. Die Bedeutung der MRT zur Diagnosestellung hat in den letzten Jahren stark zugenommen. In der T1-Wichtung lassen sich verdickte Faszien darstellen, in der T2-Wichtung eine erhöhte Signalintensität sowie eine erhöhte Kontrastverstärkung der normalerweise „dunkel“ erscheinenden Faszien in der T1-Wichtung nach Gadoliniumgabe.

Im Jahr 2014 wurden von Pinal-Fernandez diagnostische Kriterien vorgeschlagen (siehe Tab. 2). Für die Diagnose sind entweder beide Hauptkriterien oder ein Hauptkriterium zusammen mit zwei Nebenkriterien erforderlich. Das Fehlen einer systemischen Sklerose ist dabei eine wichtige Voraussetzung, da sich die Therapie wesentlich unterscheidet [2].

**Tab. 2:** Diagnosekriterien nach Pinal-Fernandez et al. [2]

Hauptkriterien	1. Symmetrische oder asymmetrische, diffuse (d. h. an den Extremitäten, am Rumpf und Bauch) oder lokalisierte (d. h. an den Extremitäten) Schwellungen, Verhärtungen und Verdickung der Haut und des subkutanen Gewebes
	2. Biopsie der gesamten Dicke der klinisch betroffenen Haut mit Faszienverdickung mit Ansammlung von Lymphozyten und Makrophagen mit oder ohne Eosinophile
Nebenkriterien	a) Periphere Eosinophilie > 0,5 × 10^9^ b) Muskelschwäche oder erhöhte Serum-Aldolase-Spiegel c) „Groove sign“ und/oder Peau-d’orange-Hautveränderungen d) Hypergammaglobulinämie e) T2-gewichtete MRT mit hyperintensen Faszien
Ausschlusskriterien	Systemische Sklerose
*Entweder sind beide Hauptkriterien oder ein Hauptkriterium und zwei Nebenkriterien für die Diagnose erforderlich*	

Im vorliegenden Fall sind beide Hauptkriterien sowie drei Nebenkriterien (periphere Eosinophilie, Hypergammaglobulinämie und MR-tomographisch hyperintense Faszien) erfüllt. Eine Systemsklerose wurde aufgrund negativer ANA serologisch ausgeschlossen; auf eine Kapillarmikroskopie wurde bei fehlendem Raynaud-Syndrom und negativen ANA verzichtet.

Die Kriterien von Pinal-Fernandez stellen eine Orientierungshilfe dar, sind jedoch bislang nicht international validiert. Die tiefe Muskelbiopsie einschließlich der Faszie gilt weiterhin als Goldstandard zur Diagnosesicherung. Typische histologische Befunde sind eine Verdickung der Faszien sowie lymphozytäre Infiltrate entlang der Faszien. Eine Eosinophilie im Gewebe kann vorhanden sein, ist jedoch nicht zwingend erforderlich für die Diagnose [1, 2].

Aufgrund der Seltenheit der Krankheit fehlen randomisierte, doppelblinde Studien zur Therapie.

Therapie der ersten Wahl sind Glukokortikoide, wobei eine initiale Pulstherapie mit Methylprednisolon die Remissionsrate erhöht und die Notwendigkeit zusätzlicher Therapien reduziert. Eine Kombinationstherapie mit Methotrexat scheint zudem effektiver zu sein als eine Monotherapie mit Prednison [1, 3].

In einzelnen Fallberichten und retrospektiven Studien wird auch der Einsatz von Rituximab, Dapson, Infliximab, Azathioprin, JAK-Inhibitoren, Immunglobulinen und Cyclosporin beschrieben [1, 5]. Eine Therapie mit Tocilizumab wurde bisher in einzelnen Kasuistiken berichtet und wird somit bisher ausschließlich „off label“ eingesetzt [4]. Tocilizumab bindet den Interleukin-6(IL-6)-Rezeptor und hemmt so die IL-6-vermittelte proinflammatorische Signalübertragung. Da IL‑6 auch die Kollagenproduktion anregt und an der Entwicklung von Fibrose beteiligt ist, könnte eine therapeutische IL-6-Hemmung z. B. mit Tocilizumab eine Rationale darstellen. Zu beachten ist, dass durch die IL-6-Hemmung die hepatische CRP-Produktion vermindert ist, sodass das CRP zur Krankheitsbeurteilung nicht mehr geeignet ist.

Das Monitoring erfolgt primär klinisch, ergänzt durch CRP, BSR und Eosinophilie. Die Aldolase hat sich als Aktivitätsmarker bewährt und kann zur Verlaufsbeurteilung benutzt werden. Bildgebend unterstützen Sonographie und MRT [5].

## Fazit für die Praxis

Wegen ihrer Seltenheit und der oft fehlenden typischen Symptome wie dem „groove sign“ oder einer Peau-d’orange-Hautveränderung ist die eosinophile Fasziitis eine häufig unterdiagnostizierte Krankheit.

- Eine eosinophile Fasziitis kann eine seltene Differenzialdiagnose darstellen bei myofaszialen Beschwerden.
- Eine systemische Sklerose muss ausgeschlossen werden: Ein Raynaud-Syndrom spricht gegen eine eosinophile Fasziitis.
- Eine Eosinophilie im peripheren Blut und in der Histologie ist nicht immer nachweisbar.
- Bei anhaltendem Verdacht sollte eine MRT der betroffenen Extremitäten durchgeführt werden.
- Systemische Glukokortikoide stellen die First-line-Therapie dar. Eine initiale Pulstherapie scheint chronische Verläufe zu verringern. Eine frühzeitige steroidsparende Therapie z. B. mit Methotrexat, ggf. mit IL-6-Blockade ist zu empfehlen.
